# Hidden in our pockets: building of a DNA barcode library unveils the first record of *Myotis alcathoe* for Portugal

**DOI:** 10.3897/BDJ.8.e54479

**Published:** 2020-07-28

**Authors:** Hugo Rebelo, Sónia Ferreira, Francisco Amorim, Pedro Horta, Helena Raposeira, Helena Santos, Pedro Beja, Vanessa A. Mata

**Affiliations:** 1 CIBIO-InBIO, Centro de Investigação em Biodiversidade e Recursos Genéticos, Universidade do Porto, Vairão, Portugal CIBIO-InBIO, Centro de Investigação em Biodiversidade e Recursos Genéticos, Universidade do Porto Vairão Portugal; 2 CIBIO-InBIO, Centro de Investigação em Biodiversidade e Recursos Genéticos, Instituto Superior de Agronomia, Lisboa, Portugal CIBIO-InBIO, Centro de Investigação em Biodiversidade e Recursos Genéticos, Instituto Superior de Agronomia Lisboa Portugal; 3 Departamento de Biologia, Faculdade de Ciências, Universidade do Porto, Porto, Portugal Departamento de Biologia, Faculdade de Ciências, Universidade do Porto Porto Portugal

**Keywords:** DNA barcoding, bats, *Myotis alcathoe*, museum collections, species identification, COI

## Abstract

**Background:**

The advent and boom of DNA barcoding technologies have provided a powerful tool for the fields of ecology and systematics. Here, we present the InBIO Barcoding Initiative Database: Portuguese Bats (Chiroptera) dataset containing DNA sequences of 63 specimens representing the 25 bat species currently known for continental Portugal. For that, we sequenced tissues samples obtained in a vast array of projects spanning the last two decades.

**New information:**

We added four new Barcoding Index Numbers (BINs) to existing Chiroptera barcodes on BOLD, two belonging to *Myotis escalerai*, one to *Plecotus auritus* and the other to *Rhinolophus hipposideros*. Surprisingly, one of the samples initially identified in the field as *Myotis mystacinus* turned out to be *Myotis alcathoe*, which represents the first record of this species for Portugal. The presence of *Nyctalus noctula* in Portugal was also genetically confirmed for the first time. This case study shows the power and value of DNA barcoding initiatives to unravel new data that may be hidden on biological collections.

## Introduction

The barcoding of life is a booming initiative to catalogue worldwide biodiversity (Ratnasingham and Hebert 2007) wherein tens of thousands of species have already been referenced through the sequencing of a fragment of the cytochrome *c* oxidase I (COI) gene. These libraries have already been used in a vast array of studies ranging from the diet assessment of species (e.g. Mata et al. 2016), to the detection of rare species (e.g. Wilcox et al. 2013) or the community composition of habitats or ecosystems (e.g. Baselga et al. 2013, Clare et al. 2007). One of the potential uses of DNA barcoding has been the taxonomic validation of biological collections (Puillandre et al. 2012). Consequently, under DNA barcoding initiatives, a great number of new species or hidden cryptic diversity have been found (e.g. Lara et al. 2010, Saitoh et al. 2015, Corley et al. 2017, Corley and Ferreira 2017). These latter examples demonstrate the power of this technique on unveiling hidden patterns of diversity.

In Portugal, the InBIO Barcoding Initiative was recently launched under the scope of EnvMetaGen ERA-Chair, aiming to contribute for building a DNA barcoding library for the country’s biodiversity of terrestrial and freshwater ecosystems (Ferreira et al. 2018). Building on this initiative, we assembled a COI reference library for all the 25 bat species known to occur in mainland Portugal (Rainho et al. 2013), belonging to four families (Vespertilionidae, Rhinolophidae, Miniopteridade and Molossidae). During this process, a new bat species was discovered for the country – *Myotis alcathoe* (Helversen et al. 2001) – raising the number of bat species for mainland Portugal to 26.

## General description

### Purpose

This dataset aims to provide a first contribution to an authoritative DNA barcode sequences library for Portuguese bats. Such a library should facilitate DNA-based identification of species for both traditional molecular studies and DNA-metabarcoding studies and constitute a valuable resource for taxonomic and ecological studies.

### Additional information

**Figure 1.**
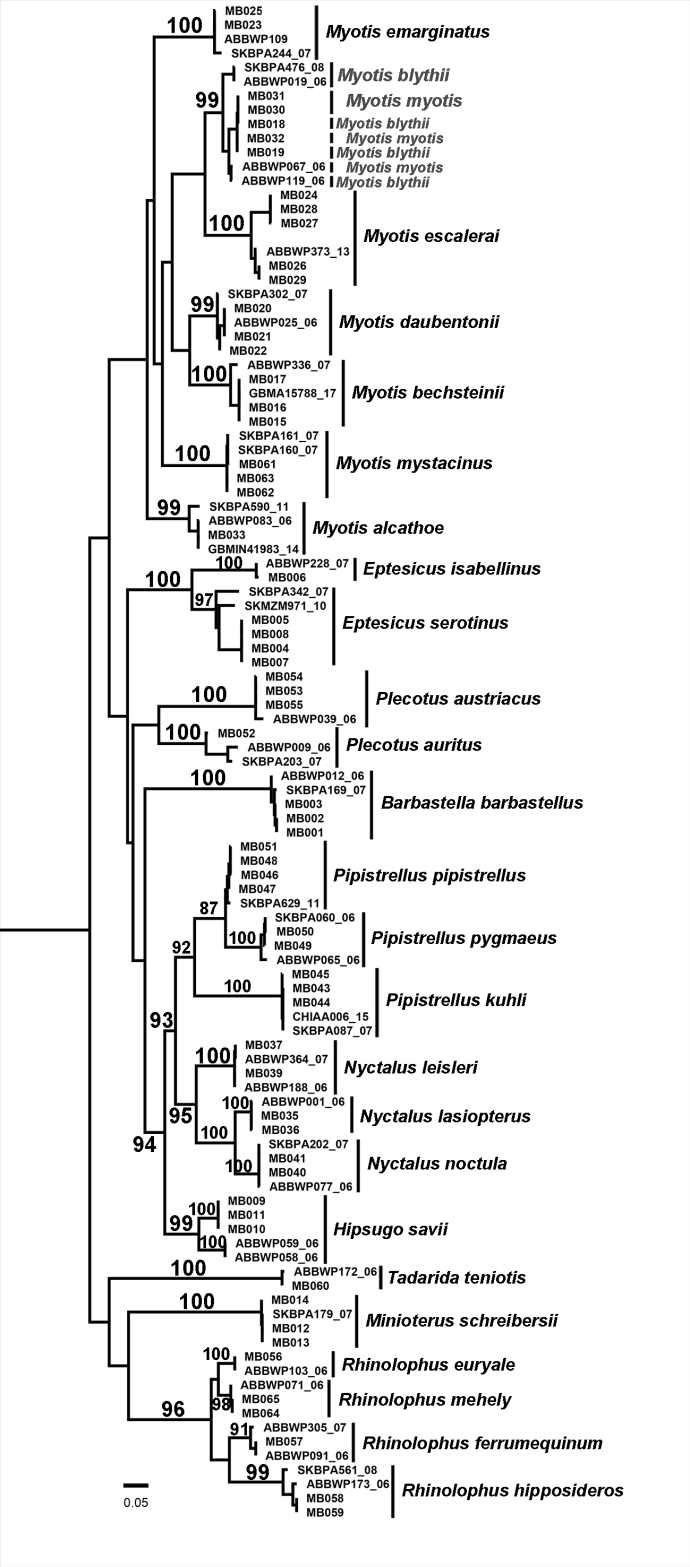
Phylogenetic tree obtained by Maximum Likelihood (ML) analysis of 26 species of bats, based on 63 newly-sequenced cytochrome c oxidase I gene (COI – 658 bp) Portuguese samples (codes started by MB) and representative sequences of all 26 species publicly available on BOLD (http://www.boldsystems.org), bootstrap values (> 80%) are indicated at nodes.

**Figure 2.**
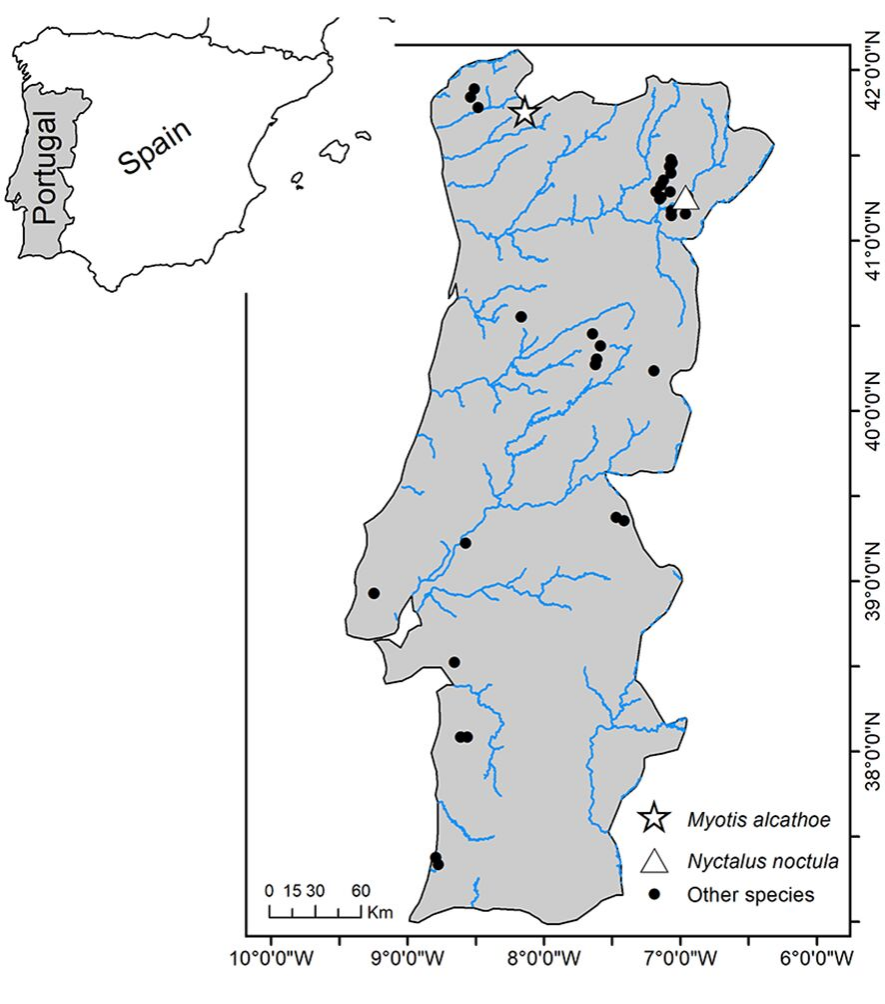
Geographic location of the analysed samples in this study.

**Figure 3.**
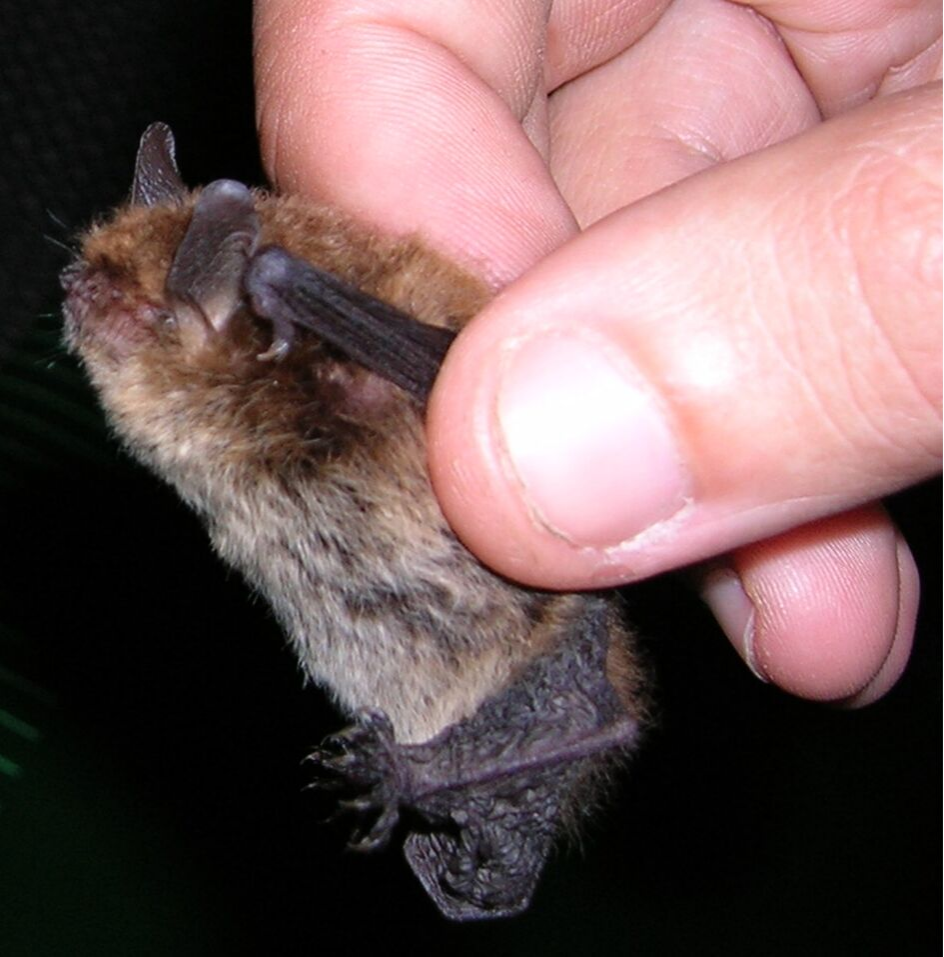
Picture of the *Myotis alcathoe* individual discovered in this study.

**Figure 4.**
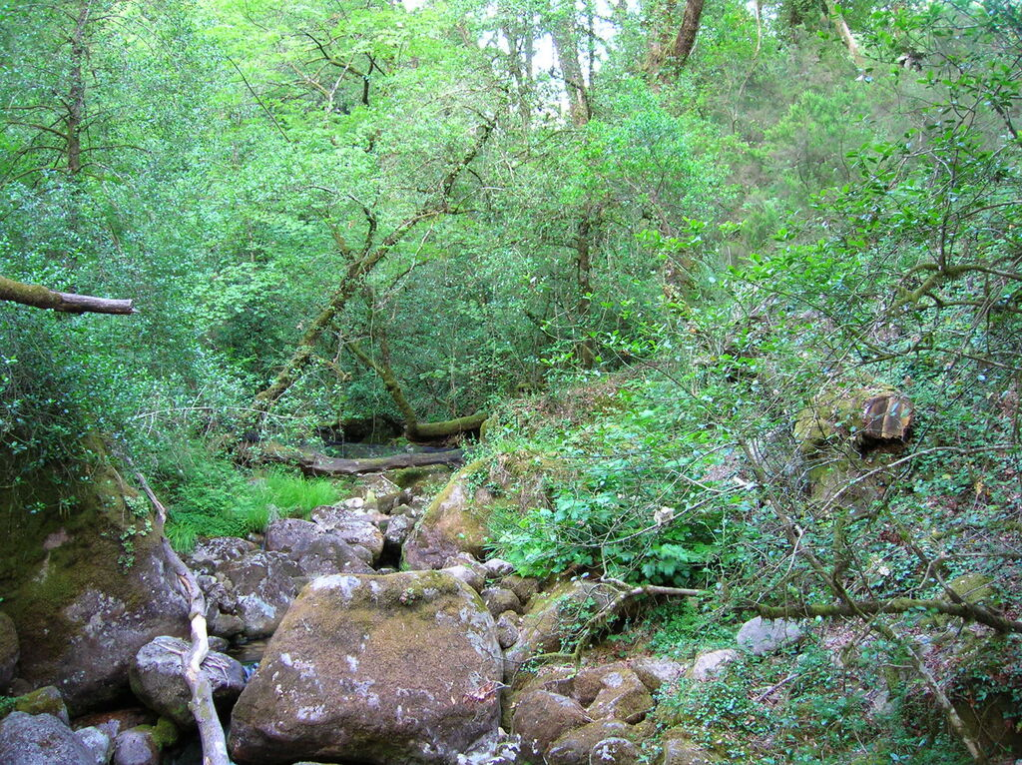
Vegetation structure of where *Myotis alcathoe* was captured.

We obtained the full barcode sequence (COI – 658 bp) for 63 specimens (Fig. 1, Table 1). Sequences are distributed in 26 Barcode Index Numbers (BINs), a system in which closely related sequences are clustered into operational taxonomic units (OTUs). Of these, four are unique, two of Myotis escalerai (ADS3148, ADT1511), one of *Plecotus auritus* (ADU1131) and one of *Rhinolophus hipposideros* (ADV3826). For five specimens, the field identification did not match the molecular data, most likely as a result of morphological misidentification (two *Eptesicus isabellinus* matched *E. serotinus* haplotypes, one *Myotis emarginatus* matched *M. escalerai*, one *M. mystacinus* matched *M. alcathoe* and one *Pipistrellus pygmaeus* matched *P. pipistrellus*). The analysed specimens of *Myotis myotis* and *M. blythii* shared the same COI haplotypes, probably due to a known widespread introgression of mtDNA (Afonso et al. 2017). It is also possible to observe the occurrence of two distinct haplogroups of *Myotis escalerai*, probably corresponding to the ‘West’ and ‘North Central East’ cytochrome-b haplogroups described by Razgour et al. (2015). Phylogenetic trees confirmed the presence of the previously known 23 bats species to mainland Portugal, plus the *M. myotis/blythii* complex (Fig. 1), as well as the occurrence of the unrecorded *M. alcathoe*. This individual was collected in a protected area located in north-western Portugal (Peneda Gerês National Park; Figs 2, 3, 4). Our results also provide the first genetic confirmation of the presence of *Nyctalus noctula* in Portugal, whose occurrence in the country consists of isolated and sporadic observation events (this study; Barros 2012, Rainho et al. 2013).

This work provides a clear example on how the DNA barcoding of collections may unveil unexpected results and reveal hidden diversity. In this case, a sample collected in 2005 and sequenced in 2018 provided the first record of *Myotis alcathoe* for Portugal. This individual was identified in the field as *M. mystacinus* that is a cryptic species of *M. alcathoe*, thus demonstrating the difficulty in distinguishing these species, based on external morphological characters alone. In fact, it was only through molecular studies that *M. alcathoe* was first identified as a separate species (Helversen et al. 2001). Yet, caution must be taken when using only mitochondrial markers for species identification, because mitochondrial introgressions have been reported for a number of species (e.g. Bogdanowicz et al. 2012). The use of these markers when introgressions are present may produce species misidentifications. Regarding the molecular identification of *M. alcathoe*, in central Europe, introgression between *M. alcathoe* and *M. mystacinus* (see Bogdanowicz et al. 2012). This way, although we cannot fully discard the possibility of the discovered female individual to be a hybrid, these authors found an overall low level of introgression, as well as an asymmetric introgression pattern mediated mainly by males, thus making it unlikely that our bat was a *M. mystacinus*. Further genetic analyses using nuclear markers would be needed to fully validate the identity of the species, but congruence of morphological characters (lighter and brown fur) and ecological ones (foraging on a very cluttered riparian gallery) seem to further support the identity of our bat individual as *M. alcathoe*.

*M. alcathoe* is associated with dense riparian environments and was known to occur from northern Spain to Sweden and Turkey. Its known distribution is highly scattered and most likely full of knowledge gaps, mainly due to under-sampling of the habitats of this bat species and misidentifications during fieldwork (Bashta et al. 2011). The closest known record of the species is separated by more than 150 km from the Portuguese sample location (Hermida et al. 2012), thus our record is also the westernmost known record for the species. Records in Spain are mainly associated with streams within mature woodlands dominated by oaks (Agirre-Mendi et al. 2004). These species’ populations are classified as Data Deficient by IUCN, with destruction and degradation of riparian forest and woodland identified as the main threats due to loss of roosts and foraging areas (Helversen et al. 2001). Of note, this species is classified as “Endangered” in Catalonia due to its rarity and pressures over riparian forests (Coronado et al. 2017). It is highly likely that the Portuguese populations may suffer from similar threats. Therefore, this species may be restricted to the northern forests of Portugal, although only through dedicated surveys will it be possible to characterise its distribution in the country and evaluate population status.

Our take-home message is that the screening of current and older collections, either museum or private, may withhold surprises that will further complete acknowledged species lists. With the ever-decreasing costs of barcoding techniques, it is expected that many researchers may afford this approach. Barcoding will most likely become an essential tool for the managing of collections. Additionally, vouchering of specimens, especially from regions with large knowledge gaps like tropical Africa and Southeast Asia, might help future studies aiming for pathogen discovery, integrative taxonomy, climate change, environmental pollution and other topics that might not constitute the initial focus of the sampling.

## Project description

### Title

The name “The InBIO Barcoding Initiative Database: Portuguese Bats (Chiroptera)” refers to the data release of DNA barcodes and distribution data of bats within the InBIO Barcoding Initiative.

### Personnel

Pedro Beja (project coordinator), Sónia Ferreira (IBI manager), Hugo Rebelo (Chiroptera specialist), Francisco Amorim (Chiroptera specialist), Pedro Horta (Chiroptera specialist), Helena Raposeira (Chiroptera specialist), Helena Santos (Chiroptera specialist), Vanessa Mata (Chiroptera specialist).

### Study area description

Continental Portugal (Fig. 2).

### Design description

Chiropteran specimens were collected in the field, morphologically identified and DNA barcoded.

### Funding

FCT - Fundação para Ciência e Tecnologia, I.P. funded V.M. (PD/BD/113462/2015) and F.A. (PD/BD/52606/2014). S.F. was funded by the project PORBIOTA - Portuguese E-Infrastructure for Information and Research on Biodiversity (POCI-01-0145-FEDER-022127), supported by Operational Thematic Program for Competitiveness and Internationalization (POCI), under the PORTUGAL 2020 Partnership Agreement, through the European Regional Development Fund (FEDER). This research also received funding from the European Union’s Horizon 2020 Research and Innovation Programme under grant agreement No 668981. Fieldwork and sample collection were funded under the scope of the FCT projects PTDC/BIA-BIC/110587/2009, LTER/BIA-BEC/0004/2009 and PTDC/BIA-ECO/31731/2017 and from EDP – Energias de Portugal.

## Sampling methods

### Study extent

Continental Portugal (Fig. 2).

### Sampling description

Bat samples were collected under the scope of several projects spanning from 2005 to 2018 (Rebelo and Jones 2010, Santos et al. 2014, Amorim et al. 2015). All bats were captured during mist-netting sessions or using harp-traps at roost exits. A non-lethal 3 mm wing punch was collected from several individuals and stored in 96% ethanol. Taxonomical identification of individuals during fieldwork was done according to the most popular identification keys of European bats (Dietz and Helversen 2004, Dietz et al. 2009).

Up to five specimens of each species were sequenced in the laboratory. DNA was extracted from wing punches, using the E.Z.N.A. Tissue Kit (Omega Bio-tek). Two partially overlapping fragments of the COI gene were amplified using the primers FwhF1 x Ind_C_R (325bp; Vamos et al. 2017, Shokralla et al. 2015) and BF2 x BR2 (423bp; Elbrecht and Leese 2017), modified to contain Illumina adaptors. PCR products were subject to a second amplification to attach indexing barcodes and P5/P7 adaptors, followed by bead clean-up, nanodrop quantification and normalisation. The final pool was quantified by qPCR and sequenced in a MiSeq platform using a v2 2x250 kit (~5000 reads/fragment/sample). Bioinformatic analysis of raw reads was done using ObiTools (Boyer et al. 2015) and, briefly, consisted of pairwise alignment of reads, removal of primer sequences, collapsing of similar reads into haplotypes and removal of rare variants (low read count). Geneious 10.2.3 (http://www.geneious.com, Kearse et al. 2012) was used for final sequence assembly, while double checking for the occurrence of possible nuclear copies. Species ID was confirmed using BOLD System Identification Platform (http://www.boldsystems.org). For each species, two representative sequences available in BOLD were retrieved and aligned with ours in order to build a phylogenetic tree. Haplotype alignments were analysed using the Maximum Likelihood (ML) method and ML trees were built in RaxML (Stamatakis 2014) with 1,000 bootstrap replicates and searching for the best-scoring ML tree.

### Quality control

All DNA barcodes sequences were compared against the BOLD database and the 99 top hits were inspected in order to detect possible issues due to contaminations or misidentifications. Prior to GBIF submission, data were checked for errors and inconsistencies with OpenRefine 3.2 (http://openrefine.org).

### Step description

Samples were collected from bats captured using mist-nets or harp-traps at roost exits and identified morphologically by experts. A non-lethal 3 mm wing punch was collected from each individual and stored in 96% ethanol from where DNA was extracted and the COI DNA barcode fragment was sequenced. Prior to GBIF submission, data were checked for errors and inconsistencies with OpenRefine 3.2 (http://openrefine.org).

## Geographic coverage

### Description

Continental Portugal (Fig. 2)

### Coordinates

37.4° and 41.9° Latitude; 9.3° and 6.9° Longitude.

## Taxonomic coverage

### Description

This dataset is composed entirely of data relating to 63 Chiroptera records.

Overall, 26 species are represented in the dataset (100% of the ones existing in continental Portugal and 83.8% of the ones existing in Iberia). These species belong to four families, the majority of which belong to the Vespertilionidae (20 species or 76.9%), with additional representatives from Rhinolophidae (four species) and a single species in the Miniopteridae and Molossidae. Vespertilionidae also accounts for over eighty percent (84.1%) of all collected samples, Rhinolophidae (9.5%), Miniopteridae (4.8%) and a single sample was collected from the Molossidae family.

### Taxa included

**Table:** 

Rank	Scientific Name	Common Name
species	*Barbastella barbastellus*	Western barbastelle
species	*Eptesicus isabellinus*	Meridional serotine
species	*Eptesicus serotinus*	Common serotine
species	*Hypsugo savii*	Savi's pipistrelle
species	*Miniopterus schreibersii*	Common bent-wing
species	*Myotis alcathoe*	Alcathoe
species	*Myotis bechsteinii*	Bechstein's
species	*Myotis blythii*	Lesser mouse-eared
species	*Myotis daubentonii*	Daubenton's
species	*Myotis emarginatus*	Geoffroy's
species	*Myotis escalerai*	Escalera's
species	*Myotis myotis*	Greater mouse-eared
species	*Myotis mystacinus*	Whiskered
species	*Nyctalus lasiopterus*	Greater noctule
species	*Nyctalus leisleri*	Lesser noctule
species	*Nyctalus noctula*	Common noctule
species	*Pipistrellus kuhlii*	Kuhl's pipistrelle
species	*Pipistrellus pipistrellus*	Common pipistrelle
species	*Pipistrellus pygmaeus*	Soprano pipistrelle
species	*Plecotus auritus*	Brown long-eared
species	*Plecotus austriacus*	Grey long-eared
species	*Rhinolophus euryale*	Mediterranean horseshoe
species	*Rhinolophus ferrumequinum*	Greater horseshoe
species	*Rhinolophus hipposideros*	Lesser horseshoe
species	*Rhinolophus mehelyi*	Mehely's horseshoe
species	*Tadarida teniotis*	European free-tailed

## Temporal coverage

**Data range:** 2005-6-27 – 2018-8-22.

### Notes

Samples were collected in the period from 27 June 2005 to 22 August 2018.

## Collection data

### Collection name

InBIO Barcoding Initiative

### Collection identifier

4ec2b246-f5fa-4b90-9a8d-ddafc2a3f970

### Curatorial unit

DNA extractions - 1 to 63

## Usage rights

### Use license

Creative Commons Public Domain Waiver (CC-Zero)

## Data resources

### Data package title

The InBIO Barcoding Initiative Database: Portuguese Bats (Chiroptera).

### Resource link

dx.doi.org/10.5883/DS-IBICH

### Number of data sets

1

### Data set 1.

#### Data set name

DS-IBICH IBI - Chiroptera

#### Data format

dwc, xml, tsv, fasta

#### Number of columns

33

#### Download URL

http://www.boldsystems.org/index.php/Public_SearchTerms?query=DS-IBICH

#### Data format version

1

#### Description

The InBIO Barcoding Initiative Database: Portuguese Bats (Chiroptera) dataset can be downloaded from the Public Data Portal of BOLD (http://www.boldsystems.org/index.php/Public_SearchTerms?query=DS-IBICH) in different formats (data as dwc, xml or tsv and sequences as fasta files). Alternatively, BOLD users can log-in and access the dataset via the Workbench platform of BOLD. All records are also searchable within BOLD, using the search function of the database.The dataset, at the time of writing the manuscript, is included as Suppl. materials 1, 2, 3 in the form of two text files for record information as downloaded from BOLD, one text file with the collecting and identification data in Darwin Core Standard format (downloaded from GBIF) and of a fasta file containing all sequences as downloaded from BOLD. It should be noted that, as the BOLD database is not compliant with the Darwin Core Standard format, the Darwin Core formatted file (dwc) that can be downloaded from BOLD is not strictly Darwin Core formatted. For a proper Darwin Core formatted file, see http://ipt.gbif.pt/ipt/resource?r=ibi_chiroptera&v=1.1 (Suppl. material 2).

All data are available in the BioStudies database (http://www.ebi.ac.uk/biostudies) under accession number **S-BSST395**.

**Data set 1.** 

Column label	Column description
processid	Unique identifier for the sample
sampleid	Identifier for the sample being sequenced, i.e. IBI catalogue number at Cibio-InBIO, Porto University. Often identical to the "Field ID" or "Museum ID"
recordID	Identifier for specimen assigned in the field
catalognum	Catalogue number
fieldnum	Field number
institution_storing	The full name of the institution that has physical possession of the voucher specimen
bin_uri	Barcode Index Number system identifier
phylum_taxID	Phylum taxonomic numeric code
phylum_name	Phylum name
class_taxID	Class taxonomic numeric code
class_name	Class name
order_taxID	Order taxonomic numeric code
order_name	Order name
family_taxID	Family taxonomic numeric code
family_name	Family name
subfamily_taxID	Subfamily taxonomic numeric code
subfamily_name	Subfamily name
genus_taxID	Genus taxonomic numeric code
genus_name	Genus name
species_taxID	Species taxonomic numeric code
species_name	Species name
identification_provided_by	Full name of primary individual who assigned the specimen to a taxonomic group
identification_method	The method used to identify the specimen
voucher_status	Status of the specimen in an accessioning process (BOLD controlled vocabulary)
tissue_type	A brief description of the type of tissue or material analysed
collectors	The full or abbreviated names of the individuals or team responsible for collecting the sample in the field
lifestage	The age class or life stage of the specimen at the time of sampling
lat	The geographical latitude (in decimal degrees) of the geographic centre of a location
lon	The geographical longitude (in decimal degrees) of the geographic centre of a location
country	The full, unabbreviated name of the country where the organism was collected
province_state	The full, unabbreviated name of the province ("Distrito" in Portugal) where the organism was collected
region	The full, unabbreviated name of the municipality ("Concelho" in Portugal) where the organism was collected
exactsite	Additional name/text description regarding the exact location of the collection site relative to a geographic relevant landmark

## Supplementary Material

488B2E2E-92D7-55DB-B20D-E690759F2238 10.3897/BDJ.8.e54479.suppl1 Supplementary material 1 IBI-Chiroptera library - Specimen details Data type Record information - specimen data Brief description The file includes information about all records in BOLD for the IBI-Chiroptera library. It contains collecting and identification data. The data are as downloaded from BOLD, without further processing. File: oo_411218.txt https://binary.pensoft.net/file/411218 Hugo Rebelo, Sónia Ferreira, Francisco Amorim, Pedro Horta, Helena Raposeira, Helena Santos, Pedro Beja, Vanessa Mata

19071C0A-9F08-5EAB-8FE1-EA9599FD5F27 10.3897/BDJ.8.e54479.suppl2 Supplementary material 2 IBI-Chiroptera library - Specimen details - Darwin Core Standard Data type Record information - specimen data in Darwin Core Standard format Brief description The file includes information about all records in BOLD for the IBI-Chiroptera library. It contains collecting and identification data. The data are downloaded from GBIF, without further processing. File: oo_411282.txt https://binary.pensoft.net/file/411282 Hugo Rebelo, Sónia Ferreira, Francisco Amorim, Pedro Horta, Helena Raposeira, Helena Santos, Pedro Sousa, Pedro Beja, Vanessa Mata

0A14D044-0419-5977-82DC-3E3A198DFFEE 10.3897/BDJ.8.e54479.suppl3 Supplementary material 3 IBI- Chiroptera library - DNA sequences Data type Genomic data, DNA sequences Brief description COI sequences in fasta format. Each sequence is identified by the BOLD ProcessID, species name, marker and GenBank accession number, separated by pipe. The data are as downloaded from BOLD. File: oo_411203.txt https://binary.pensoft.net/file/411203 Hugo Rebelo, Sónia Ferreira, Francisco Amorim, Pedro Horta, Helena Raposeira, Helena Santos, Pedro Beja, Vanessa Mata

## Figures and Tables

**Table 1.** List of species that were collected and DNA barcoded within this project. * Indicate species with new BINs.

**Family**	**Species**	**IBI code**	**BOLD code**	**BOLD BIN**	**GenBank**
Miniopteridae	*Miniopterus schreibersii*	MB12	IBICH012-19	AAC3658	MT407281
		MB13	IBICH013-19	AAC3658	MT407282
		MB14	IBICH014-19	AAC3658	MT407283
Molossidae	*Tadarida teniotis*	MB60	IBICH056-19	AAB2570	MT407332
Rhinolophidae	*Rhinolophus euryale*	MB56	IBICH052-19	AAF7222	MT407326
Rhinolophidae	*Rhinolophus ferrumequinum*	MB57	IBICH053-19	AAD7131	MT407327
Rhinolophidae	Rhinolophus hipposideros*	MB58	IBICH054-19	ADV3826	MT407328
		MB59	IBICH055-19	ADV3826	MT407329
Rhinolophidae	*Rhinolophus mehelyi*	MB64	IBICH060-19	AAF7233	MT407330
		MB65	IBICH061-19	AAF7233	MT407331
Vespertilionidae	*Barbastella barbastellus*	MB01	IBICH001-19	AAF0184	MT407270
		MB02	IBICH002-19	AAF0184	MT407272
		MB03	IBICH003-19	AAF0184	MT407271
Vespertilionidae	*Eptesicus isabellinus*	MB06	IBICH006-19	AAX8557	MT407277
Vespertilionidae	*Eptesicus serotinus*	MB04	IBICH004-19	AAC2865	MT407276
		MB05	IBICH005-19	AAC2865	MT407273
		MB07	IBICH007-19	AAC2865	MT407274
		MB08	IBICH008-19	AAC2865	MT407275
Vespertilionidae	*Hypsugo savii*	MB09	IBICH009-19	AAC2816	MT407279
		MB10	IBICH010-19	AAC2816	MT407278
		MB11	IBICH011-19	AAC2816	MT407280
Vespertilionidae	*Myotis alcathoe*	MB33	IBICH031-19	AAF5058	MT407284
Vespertilionidae	*Myotis bechsteinii*	MB15	IBICH015-19	AAD0964	MT407285
		MB16	IBICH016-19	AAD0964	MT407287
		MB17	IBICH017-19	AAD0964	MT407286
Vespertilionidae	*Myotis blythii*	MB18	IBICH018-19	AAC9255	MT407289
		MB19	IBICH019-19	AAC9255	MT407288
Vespertilionidae	*Myotis daubentonii*	MB20	IBICH020-19	AAA8808	MT407290
		MB21	IBICH021-19	AAA8808	MT407292
		MB22	IBICH022-19	AAA8808	MT407291
Vespertilionidae	*Myotis emarginatus*	MB23	IBICH062-19	AAD0937	MT407294
		MB25	IBICH063-19	AAD0937	MT407293
Vespertilionidae	Myotis escalerai*	MB24	IBICH023-19	ADT1511	MT407297
		MB26	IBICH024-19	ADS3148	MT407298
		MB27	IBICH025-19	ADT1511	MT407299
		MB28	IBICH026-19	ADT1511	MT407296
		MB29	IBICH027-19	ADS3148	MT407295
Vespertilionidae	*Myotis myotis*	MB30	IBICH028-19	AAC9255	MT407302
		MB31	IBICH029-19	AAC9255	MT407301
		MB32	IBICH030-19	AAC9255	MT407300
Vespertilionidae	*Myotis mystacinus*	MB61	IBICH057-19	AAB4668	MT407303
		MB62	IBICH058-19	AAB4668	MT407304
		MB63	IBICH059-19	AAB4668	MT407305
Vespertilionidae	*Nyctalus lasiopterus*	MB35	IBICH032-19	AAF3011	MT407306
		MB36	IBICH033-19	AAF3011	MT407307
Vespertilionidae	*Nyctalus leisleri*	MB37	IBICH034-19	AAC4752	MT407308
		MB38	IBICH035-19	AAC4752	MT407310
		MB39	IBICH036-19	AAC4752	MT407309
Vespertilionidae	*Nyctalus noctula*	MB40	IBICH037-19	AAC7411	MT407311
		MB41	IBICH038-19	AAC7411	MT407312
Vespertilionidae	*Pipistrellus kuhlii*	MB43	IBICH039-19	AAA7926	MT407313
		MB44	IBICH040-19	AAA7926	MT407314
		MB45	IBICH041-19	AAA7926	MT407315
Vespertilionidae	*Pipistrellus pipistrellus*	MB46	IBICH042-19	AAC5524	MT407316
		MB47	IBICH043-19	AAC5524	MT407317
		MB48	IBICH044-19	AAC5524	MT407319
		MB51	IBICH047-19	AAC5524	MT407318
Vespertilionidae	*Pipistrellus pygmaeus*	MB49	IBICH045-19	AAB4312	MT407321
		MB50	IBICH046-19	AAB4312	MT407320
Vespertilionidae	Plecotus auritus*	MB52	IBICH048-19	ADU1131	MT407322
Vespertilionidae	*Plecotus austriacus*	MB53	IBICH049-19	AAD0926	MT407323
		MB54	IBICH050-19	AAD0926	MT407324
		MB55	IBICH051-19	AAD0926	MT407325
